# Divergent selection for litter size variability affects RNA cargo in oviductal extracellular vesicles related to embryonic development and survival

**DOI:** 10.1186/s40659-025-00642-1

**Published:** 2025-09-26

**Authors:** Carmen Almiñana, María-José Argente, Daniel Serrano-Jara, Meriem Hamdi, Stefan Bauersachs, María-Luz García

**Affiliations:** 1 Institute of Veterinary Anatomy, Vetsuisse Faculty, University of Zurich, Lindau, Zurich, Switzerland; 2 Department of Reproductive Endocrinology, University Hospital Zurich, Zurich, Switzerland; 3 Departamento de Tecnología Agroalimentaria, Instituto de Investigación e Innovación Agroalimentaria y Agroambiental (CIAGRO-UMH, Universidad Miguel Hernández de Elche, Orihuela, Spain

**Keywords:** Oviduct, Extracellular vesicles, Rabbit, Doe, Pregnant, Embryo, RNA-sequencing, Litter size, Divergent selection

## Abstract

**Background:**

Selection for increased homogeneity of litter size has been proposed to improve resistance to stress and diseases in animals. Previous studies have shown that lines selected for decreased litter size variability (L lines) have higher litter sizes at parity than lines selected for increased litter size variability (H lines), possibly due to higher embryo survival in the oviduct. Thus, the study aimed at examining the effect of the line selection on the oviduct environment and its contribution to embryo development, particularly via extracellular vesicles (EVs). Oviductal EVs (oEVs) and their molecular cargo play essential roles in supporting early embryo development in different species, but little is so far known in rabbits.

**Results:**

Oviductal fluid was collected by flushing oviducts from pregnant (with embryos at 72 h p.c., P) and control does (non-ovulated/non-pregnant, NO) from the two lines, resulting in 4 experimental groups: pregnant H line (H_P); pregnant L line (L_P); non-pregnant H line (H_NO); non-pregnant L line (L_NO). Oviductal EVs were isolated and characterized. RNA cargo of oEVs was analyzed by RNA-sequencing, revealing a high number of differential abundant (DA) genes between P vs. NO does in both lines (1223 DA genes in H line and 1519 in L line, FDR < 0.1%). Additionally, 27 and 25 miRNAs were found as DA between P vs. NO in H and L lines, respectively. Interestingly, functional enrichment analysis of DA genes and predicted target genes of identified miRNAs revealed biological terms such as embryo development, epithelium morphogenesis and differentiation, and cilium movement, which were only associated to L line for P and NO groups. Furthermore, the comparison between H and L lines identified 169 DA RNAs in NO does, but without significant differences in P does. For miRNAs, no differences were detected in H vs. L lines in P or NP does.

**Conclusions:**

This is the first study unveiling the differential oEV RNA cargo between lines selected for low versus high variation in litter size, and in each line, between P and NO does. The changes in protein-coding RNA and miRNA cargo might reflect the different maternal support to the early embryo development in the different lines.

**Supplementary Information:**

The online version contains supplementary material available at 10.1186/s40659-025-00642-1.

## Background

Litter size is an important economic trait in prolific species and has been the objective of different selection programs to produce maternal lines [1]. However, these genetically selected lines tend to be particularly sensitive to stress and diseases in pigs [2] and in rabbits [3, 4]. In rabbits, a divergent selection experiment for environmental sensitivity has been carried. Environmental sensitivity has been measured as the phenotypic variance of litter size at birth within each female. Two lines were developed: The L line is selected for reducing litter size variability and the H line is selected for increasing litter size variability [5]. The L line does exhibits a lower inflammatory response to infections, greater disease resistance and less stressed compared to the H line [6, 7]. These results are consistent with the higher fertility, lower mortality at parturition of females, the lower percentage of litter mortality at birth and at weaning, and the higher homogeneity of litter weight at weaning found in the L line [6]. In short, L line is more resilient than H line [6, 7]. Additionally, the L line has a larger litter size [8], as a consequence of a more advanced embryonic development in the first hours of gestation [9]. The main part of the pre-implantation embryo mortality occurs before 72 h of gestation, when the embryos are in the oviduct [10].

The mechanisms underlying the relationship between stress and reproduction have been studied in different species (in pigs [11], in guineapigs [12] and in chicken [13]). Stress is commonly linked to enhanced activity of the hypothalamo-pituitary-adrenal (HPA) axis and the activation of the sympathetic adreno-medullary system. Activation of the HPA system results in the secretion of peptides from the hypothalamus, principally corticotropin-releasing hormone, which stimulates the release of adrenocorticotropic hormone (ACTH) and beta-endorphin. ACTH induces the secretion of corticosteroids from the adrenal cortex, which can be seen in animals exposed different stressors [11]. High doses of ACTH cause a delay in the onset of oestrus and development of cystic follicles [14], a delayed embryonic cleavage rate, and decreased number of spermatozoa attached to the zona pellucida, reflecting in the oviductal environment in sows [15, 16]. We hypothesize that the elevated stress levels observed in H line rabbits may impact various stages of gestation, with particular sensitivity noted in the oviductal environment. Therefore, understanding the oviductal environment is essential to elucidate the differences in embryo development and survival between the lines.

Extracellular vesicles (EVs), are nanosized membrane-enclosed vesicles, containing diverse molecular cargo (mRNAs, small non-coding RNAs, proteins, lipids, metabolites and DNA) that can be shuttled to different recipient cells and change the phenotype of the recipient cell [17, 18]. The EVs are present in male seminal fluid and female reproductive fluids such as follicular, uterine or oviductal fluid (reviewed in [19]). Among them, the EVs in the oviductal fluid have raised increasing interest in the recent years as key components of the oviductal environment in different species: bovine, mouse, porcine, canine, feline, even avian or turtle (reviewed in [20]) and significant contributors to the early reproductive events [20–23]: from gametes maturation [24], sperm motility and survival [25], fertilization [26] and embryo development [27–29]. Besides, their molecular cargo (RNAs, proteins or metabolites) has been shown to be modulated by the estrous cycle or the pregnancy status in different species [27, 28, 30–32]. The use of oEVs as potential tools to improve in vitro embryo production (IVP) resulted in an enhancement of blastocyst rates and changes in the embryonic transcriptome [33, 34]. Altogether, it points them as modulators of gamete/embryo-maternal interactions and boosters of early embryo development and growth.

However, little is known about the oEVs in rabbits (*Oryctolagus cuniculus*). The rabbit has been considered a classic laboratory animal model, used as an experimental reference for other species for studies on embryology [35, 36], studies on the effect of assisted reproductive techniques [37], and for specific diseases and their effect on subsequent generations [38–40]. Other particularities of the female reproductive physiology make the rabbit an interesting study object for early reproductive events since the rabbit is a species in permanent estrus [41] or undergoing periods of behavioral estrus [42], with induced ovulation by mating in contrast to other mammals. On the other hand, the rabbit is a species with an increasing economic value being a source of healthy and highly nutritional meat [43] and the third species in terms of number of animals reared for meat production in the world (source: FAOSTAT. *Food and Agriculture Data*. Food and Agriculture Organization of the United Nations, 2020: Available online at: http://www.fao.org/faostat/en/#data).

Considering the differences found in embryo development of divergent selected lines for litter size variability, together with the lack of knowledge about oEVs and their potential contribution to the oviductal environment, the objectives of the present study were: (1) to identify and characterize the rabbit oEVs; (2) to unveil differences in oEV secretion and RNA cargo in pregnant versus non-pregnant does and; (3) to determine differences in oEVs cargo between two lines divergently selected for litter size variability.

## Methodology

### Animals

All experimental procedures involving animals were approved by the Miguel Hernández University of Elche Research Ethics Committee (Reference number 2019/VSC/PEA/0017), in accordance with the International Guiding Principles for Biomedical Research Involving Animals, as promulgated by the Society for the Study of Reproduction and EU Directive 2010/63/EU.

Females came from generation 12 of a divergent selection experiment for litter size variability [5]. Each divergent line had approximately 125 females and 25 males per generation. The selection criterion was litter size variability at birth. Variability of litter size was estimated as phenotypic variance of litter size at birth within female considering all parities, after correcting litter size for the effects of year-season and parity-lactation status. The average of the litter size variability was 2.27 (± 1.97) and 3.84 (± 3.69) for low and high lines at generation 12, respectively.

Females were housed at the Miguel Hernández University of Elche farm in individual wire cages. Animals were kept under a controlled 16-h light: 8-h dark photoperiod and were fed a commercial diet.

### Experimental design

A total of 8 multiparous, non-lactating females from the L line, selected for homogeneity in litter size (i.e., low variance), and 8 multiparous, non-lactating females from the H line, selected for heterogeneity in litter size (i.e., high variance), were used in the experiment. In total, samples of oviductal fluid from 16 does were collected from 4 experimental groups with 4 animals per experimental group (4 biological replicates, *N* = 4). Samples were labelled as follows: rabbit line (L or H), pregnancy status (P or NO) and no. of doe (Table 1).

### Oviductal fluid, embryo recovery and sample processing

Litter size of the first 4 parities was recorded in all females and the mean and variance of the litter size was calculated (Table 1). At the end of the 4th lactation, 8 females (4 from each line) were mated with males with proven fertility to obtain embryos (pregnant group) and 8 females were not mated, so they did not ovulate (non-pregnant group). Then, the 16 females were slaughtered by intravenous (i.v.) injection of sodium thiopental at 50 mg/kg body weight (Tiobarbital^®^, B. Braun Medical S. A., Barcelona, Spain) at 72 h post-coitum (hpc). The entire reproductive tract was removed after slaughter. The oviducts were excised, and each oviduct was flushed once with 5 mL of Dulbecco´s Phosphate Buffered Saline (^®^DPBS, Sigma, Alcobendas, Madrid, Spain) supplemented with anhydrous CaCl_2_ (0.132 g/L) and 2 g/L of bovine serum albumin (^®^BSA, Sigma, Alcobendas, Madrid, Spain) at room temperature.

In pregnant group, corpora lutea were counted. Embryos were recovered from the oviductal fluid, counted, and classified using a binocular stereoscopic microscope (Leica MZ75-200x). Embryo classification was performed based on their morphology: normal embryos when they presented homogenous cellular mass and intact zona pellucida [44] and abnormal the rest. Normal embryos were classified as early morulae and compacted morulae.

**Table 1:** Description of the experimental groups

Sample_Code	Line	Pregnant(*P*)/ Non-Pregnant (NO)	Litter Size Average (kits)	Litter Size Variance (kits^2^)*
L_P_1	L	*P*	8.3	3.9
L_P_5	L	*P*	8.1	3.3
L_P_9	L	*P*	10.5	1.9
L_P_13	L	*P*	10.9	4.8
L_NO_2	L	NO	7.2	4.7
L_NO_6	L	NO	7.4	0.3
L_NO_10	L	NO	7.2	6.7
L_NO_14	L	NO	7.0	2.0
H_P_3	H	*P*	7.2	6.5
H_P_7	H	*P*	6.0	13.5
H_P_11	H	*P*	7.4	4.3
H_P_15	H	*P*	7.4	4.5
H_NO_4	H	NO	5.8	7.8
H_NO_8	H	NO	7.7	10.1
H_NO_12	H	NO	7.0	7.6
H_NO_16	H	NO	8.0	3.9

*$\:{kits}^{2}=\frac{1}{n+1}\sum\:_{1}^{n}{(xi-\stackrel{-}{x})}^{2}$; where n is the total number of parities of the doe, and $\:xi$ is litter size of a doe’s parity $\:i$

After embryo recovery, oviductal flushing was collected and processed. All oviductal fluid samples were subjected to serial centrifugation. First, oviductal fluid samples were centrifugated at 300 *g* for 15 min at 4 °C to remove the cells. The supernatant was transferred to a new tube and centrifuged at 2000 *g* for 15 min at 4 °C to remove cellular debris. Subsequently, supernatant samples were frozen and stored at − 80 °C.

### Isolation of rabbit oEVs

Frozen supernatant samples from all does were transported on dry ice to the laboratories of the University of Zurich. The protocol used for isolation of rabbit oEVs was used previously to successfully isolate EVs from different female reproductive fluids: follicular [45], uterine [46, 47] and oviductal [25–28, 30] in bovine, porcine and equine. Briefly, all OF samples were thawed on ice and centrifuged at 12,000 *g* for 30 min at 4 °C to remove cellular debris, apoptotic bodies and bigger microvesicles. The pellet obtained after 12,000 *g* was suspended in PBS (referred as large EVs) and stored for further examination. The supernatant was used for subsequent EVs isolation by ultracentrifugation (UC) at 100,000 *g* for 90 min at 4 °C (with swinging bucket Beckman rotor MLS-50, Beckman tubes Ultra-clear, No.344057; filled with PBS-Trehalose (PBS: #P4417-100TAB, Sigma-Aldrich Chemie GmbH and 25 mM trehalose, Sigma, T0167, Sigma-Aldrich Chemie GmbH) to 5 ml and the use of the Beckman Optima MAX-XP ultracentrifuge (Beckman Coulter International S.A.). The first UC step followed a second UC with the same parameters to wash the EVs pellet with PBS-Trehalose. Finally, pellets after the second UC from each OF were carefully suspended in 50 µl PBS-Trehalose 25 mM µl [46] and aliquots were snap frozen and stored at -80 °C for subsequent characterization experiments and analysis of RNA content by RNA-sequencing.

### Characterization of rabbit oEVs

#### Analysis of oEVs by transmission electron microscopy (TEM)

For TEM observations, EVs suspensions were diluted in PBS and fixed in glutaraldehyde (freshly prepared) (1% final concentration). Three microliters of each EVs sample were placed on the formvar carbon-coated grid for 5 min and washed with distilled water (three times). For negative contrast the samples were incubated in 2% water solution of uranyl acetate (30 s three times, 5 µl) and left to dry in the small drop (near 1 µl) of last solution. The micrographs were obtained using TEM HITACHI HT 7700 Elexience at 80 kV (with a charge-coupled device camera AMT) and JEM 1011 (JEOL, Japan) equipped with a Gatan digital camera driven by Digital Micrograph software (Gatan, Pleasanton, USA) at 100 kV. For TEM analysis, 4 pools of EVs samples (each pool containing 1 µl of sample of each of the 4 biological replicates in each experimental group, 4 µ/pool) were analyzed by TEM.

#### Analysis of oEVs size distribution and concentration by nanoparticle tracking analysis

Nanoparticle tracking analysis (NTA) was carried out on a NanoSight NS300 (Malvern Panalytical, Westborough, MA, USA) embedded with laser: 45 mW at 488 nm and an automated syringe sampler. EV samples were diluted 1:1000–1:10000 in PBS and loaded into 1 ml syringes with Syringe Pump speed of 50 µL/s and 24.6–24.7 °C temperature. For each measurement, five 1-min videos were captured under the following conditions: sCMOS camera, camera level 8. After capture, the videos were analyzed by the in-build NanoSight Software NTA 3.1 Build 3.1.46 with a detection threshold of 3. Autofocus was adjusted so that indistinct particles were avoided. Four biological replicates for each experimental group were analyzed by NTA and measurements of mean particle size, mode and concentration particles/ml were performed (16 samples).

#### Protein quantification and Western blotting

Measurements of protein concentration in all preparations of large EVs (obtained after 12,000 *g*) and small EVs (obtained after second UC 100,000 *g*) were performed using the Pierce™ BCA Protein Assay (Pierce™ BCA Protein Assay Kit, ThermoFisher Scientific), according to the manufacturer’s instructions.

To characterize the rabbit oEVs with known exosomal markers by Western blotting, proteins from pools of large and small EVs samples were first separated by gradient Sodium Dodecyl Sulphate-Polyacrylamide gel electrophoresis (SDS-PAGE) in a 4 to 20% polyacrylamide gel (Stain-free gel, #4568093, Bio-Rad Laboratories AG). After SDS-PAGE, proteins profiles were visualized by ChemiDoc MP Imaging System (Stain free blots, Bio-Rad Laboratories AG). Then, proteins from large and small EVs samples were transferred to nitrocellulose protean membranes (Trans-Blot Turbo Transfer Mini Nitrocel. membrane, Biorad, 170–4158) with a Trans-Blot Turbo Transfer System (BioRad, program mixed, 7 min, transfer). The transfer was followed by 1 h membrane incubation with blocking solution of 5% skim milk (Sigma 70166) in TBS-Tween 0.05% (TBT; BioRad, 1706435 and Tween; Sigma P9416) (TBS-T). Incubation of membranes with primary antibodies diluted in blocking solution (TBS-T milk 5%) was performed overnight at 4 °C. Then, the membranes were washed with TBS-T three times, 10 min each, before the incubation with secondary antibodies diluted in TBS-T for 1 h at room temperature. Antibodies and dilutions used for Western Blotting experiments were as follow: For primary antibodies, Anti-CD9 Mouse Monoclonal Antibody, Clone MM2/57, MCA469GT, Bio-Rad, 1:500; Anti-ALIX Mouse Monoclonal Antibody Santa Cruz sc-53,540, 1:500; Anti-TSG101 Rabbit Polyclonal Antibody, PA5-31260 Invitrogen, 1:1000 were used. For secondary antibodies, Anti-mouse m-IgGκ BP-HRP Santa Cruz sc-516,102, 1:10000; goat anti-rabbit IgG-HRP Santa Cruz sc-2004, 1:8000 were used. Subsequently, the membranes were washed with 5 ml of TBS-T three times, 15 min each, before developing the immune blot with the Clarity Max Western Blotting ECL Substrates (BioRad 170–5062). ChemiDoc MP Imaging System was used to detect proteins after Western blot (Bio-Rad).

### Analysis of RNA cargo of rabbit oEVs by RNA-sequencing

#### RNA isolation, RNA quantification and assessment of RNA quality

The total RNA from 16 oEV samples was isolated using the miRNeasy micro kit (QIAGEN AG, Hombrechtikon, Switzerland) according to the manufacturer’s instructions. RNA concentration was measured by different RNA quantification methods: Agilent RNA 6000 Pico assay (Agilent 2100 Bioanalyzer, Agilent Technologies Schweiz AG, Basel, Switzerland) for RNA quantity and quality profiles of EVs samples; and Quantus™ Fluorometer (Promega AG, Dübendorf, Switzerland) together with QuantiFluor RNA System kit (Promega) for RNA concentration. In total, 16 libraries were prepared and used for RNA-sequencing.

#### Low-input total RNA library preparation and sequencing

RNA-Seq library preparation was performed by using the SEQuoia Complete Stranded RNA Library Prep Kit (Bio-Rad Laboratories, Inc. Cressier, Switzerland), which permits the capture of long as well as short RNAs in a single library. A total of 3 ng total RNA was used for EV sample. One pool of the 16 samples was prepared, and sequencing was performed on one SP flow cell on an Illumina NovaSeq 6000 instrument (Functional Genomic Center Zurich). Paired-end sequencing was performed with 92 bp for read one (cDNA insert) and 8 bp for read 2 (UMI sequence for removal of PCR duplicates).

#### RNA-seq data analysis

Data analysis was performed on a locally installed version of Galaxy [48]. Sequencing reads were processed using Cutadapt (Galaxy version 1.16.8) with the parameters -u 1 (trim first base at 50), -a A(10) (trim any poly(A) track and following bases in the read), -m 15 (removes reads shorter than 15 bases), and a quality cutoff of 28. Trimmed reads were mapped to the rabbit genome reference assembly OryCun2.0 with HISAT2 (Galaxy version 2.1.0 + galaxy4). NuDUP mark/remove PCR duplicates based on molecular tags (Galaxy version 2.3.3) was used to remove PCR duplicates from the BAM files before counting reads mapped to the annotated features of the porcine genome with the tool featureCounts (Galaxy version 1.6.4 + galaxy1) based on the NCBI genome annotation file (GCF_000003625.3_OryCun2.0_genomic.gff).

A separate counting was performed for reads mapping to mature microRNAs (miRNAs) with the MiRDeep2 Quantifier (Galaxy version 2.0.0) based on miRNA sequences of miRBase (version 22.1). MicroRNAs that showed at least 1 count in at least 1 sample were identified as detected miRNAs. MicroRNAs that showed at least 10 counts in at least 3 samples of the same group (in at least one group) were selected for further differential expression analysis.

Further analysis was performed in R with the BioConductor package EdgeR [49] to identify differentially expressed genes (DEGs) and differentially abundant (DA) miRNAs. Data normalization was performed using TMM normalization and GLMRobust generalized linear model [49]. DEGs and DA miRNAs were defined based on the false discovery rate (FDR).

### Data mining and bioinformatics analysis of RNA EVs cargo

Gene symbols and Entrez Gene IDs (Oryctolagus cuniculus) were mapped for all transcripts, using bioinformatics custom tools integrated in a local Galaxy installation. Clustering analyses were performed using Multiple Experiment Viewer tool (MeV v.4.8.1, https://sourceforge.net/projects/mev-tm4/) to created HCL and SOTA expression images [50]. Target gene analysis of identified miRNAs was performed using MIENTURNET webtool (database miRTarBase) (http://userver.bio.uniroma1.it/apps/mienturnet) [51]. To identify enriched functional terms for genes or target genes of miRNAs identified in oEV, Metascape online tool (www.metascape.org) [52] and DAVID functional annotation clustering were used (https://david.ncifcrf.gov/) [53]. To compare DA genes, miRNAs and their target genes contained in oEVs from different experimental groups, Jvenn, an integrative tool for comparing lists of genes with Venn Diagrams was used [54]. Finally, comparative enrichment clustering and network visualization of DA genes or target genes was performed with ToppCluster (https://toppcluster.cchmc.org/publications.jsp) [55]. Cytoscape was used to improve the network generated by Toppcluster and provided a final clear image of the gene network [56].

### Data availability

RNA-Seq data have been deposited at NCBI’s Sequence Read Archive (SRA) under the BioProject accession PRJNA1209703 (http://www.ncbi.nlm.nih.gov/bioproject/1209703).

### Statistical analysis

Ovulation rate, embryo recovery, normal embryo, number of oocytes, percentage of early and compacted morulae, and litter size average and variability were analyzed with a model including the effect of line (H and L lines). The traits were analyzed using Bayesian methodology. Bounded flat priors were used for all unknowns. Residuals were normally distributed with mean 0 and variance Iσ^2^_e_. The priors for the variances were also bounded uniform. Features of the marginal posterior distribution of differences between lines were estimated using Gibbs sampling. The Rabbit program developed by the Institute for Animal Science and Technology was used for all procedures. Inferences were made from the estimated marginal posterior distributions of the differences between the H and the L lines [57].

Concentration of particles, mean of EV size and EV RNA concentrations are presented as the mean ± SEM. The variables in all experiments were tested for their normality (Shapiro-Wilk test) before being analyzed by one-way analysis of variance (ANOVA) followed by Tukey’s test. Two-sided *P =* 0.05 was considered significant. Statistical analysis was performed by using Prisma 8 program, version 8.2.0. (GraphPad Software, San Diego, CA, USA) (https://www.graphpad.com/scientific-software/prism/).

## Results

### Embryo recovery and classification

Features of the estimated marginal posterior distributions of the differences between lines H and L for corpora lutea, embryo development, and litter size are presented in Table 2. This table reports the probability that these differences are greater than zero when H–L > 0, or less than zero when H–L < 0. It is important to note that, in a Bayesian framework, there is no concept of “statistical significance” in the classical sense; instead, the actual probabilities of the differences being greater or smaller than zero are directly estimated. Furthermore, in Bayesian analysis, these probabilities can reach or exceed 0.90 even when the 95% credible intervals include zero [57]. No differences were observed between does from H and L lines for number of corpora lutea counted per female, percentage of embryo collected, and percentage of normal embryo (*P* < 90%; Table 2). Besides, the number of oocytes recovered was higher in the H line than in the L line (+ 0.50 oocytes; *P* = 90%). The collected normal embryos were classified in early morula, and compacted morula, showing 56.2% early morulae the H line and 24.2% the L line (*P* = 79%). By contrast, 43.8% and 75.8% compacted morulae were found in the H and L lines, respectively (*P* = 79%). Differences between H and L lines were observed for litter size average (H line: 7.06 kits and L line: 8.33 kits; *P* = 96%) and variance (H line: 6.13 kits^2^ and L line: 3.45 kits^2^; *P* = 94%).

**Table 2:** Differences between lines for corpora lutea, embryo development and litter size

	H	L	H-L	HPD_95%_	*P* (%)
No. Corpora Lutea	11.75	11.76	-0.01	-4.47; 4.95	51
Embryos Recovery (%)	76.27	55.76	20.51	-55.35; 89.93	75
Normal Embryos (%)	87.33	99.95	-12.62	-52.96; 29.89	77
Early Morulae (%)	56.16	24.23	31.93	-22.84; 99.21	79
Compacted Morulae (%)	43.84	75.77	-31.93	-97.32; 24.65	79
No. Oocytes	0.50	0.00	0.50	-0.39; 1.48	90
Litter Size Average (kits)	7.06	8.33	-1.27	-2.71; 0.16	96
Litter size Variance (kits^2^)*	6.13	3.45	2.68	-0.98; 5.9	94

H = median of the high line; L = median of the low line; H-L = median of the difference between the high and the low lines; HPD95% = highest posterior density region at 95%; *P* = probability of the difference being > 0 when H-L > 0, and probability of the difference being < 0 when H-L < 0

*$\:{kits}^{2}=\frac{1}{n+1}\sum\:_{1}^{n}{(xi-\stackrel{-}{x})}^{2}$; where n is the total number of parities of the doe, and $\:xi$ is litter size of a doe’s parity $\:i$

### Characterization of rabbit oEVs

**Fig. 1:**
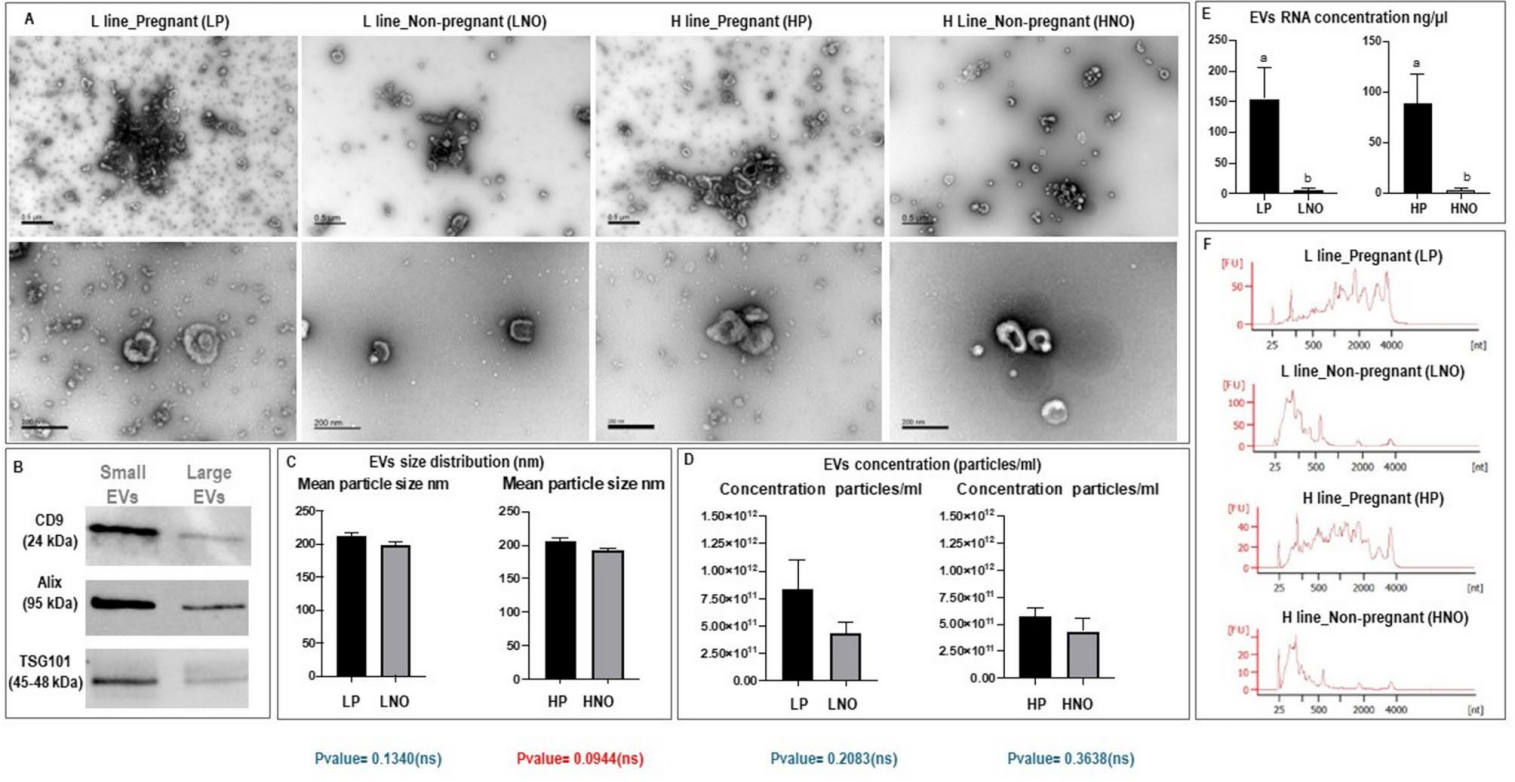
Characterization of rabbit oviductal extracellular vesicles (oEVs). (**A**) Representative images (transmission electron microscopy) of oEVs samples isolated from oviductal fluid samples from pregnan (P) and non-pregnant (NO) does from two lines with high litter size variability (H) compared to low litter size variability (L). (**B**) Western blot characterization of small EVs (100,000 g) and large EVs (after 12,000 x *g* pellet) for known exosomal protein markers CD9, ALIX and TSG101. **C** and **D**) Comparison of oEVs size distribution and particle concentration across samples measured by nanoparticle tracking analysis (NanoSight NS300). **E** and **F**) Comparison of oEVs RNA concentration and RNA profiles across samples measured by Quantus™ Fluorometer and by Agilent 2100 Bioanalyzer, respectively

Transmission electron microscopy (TEM) observations confirmed the presence of EVs in oviductal fluid of the four experimental groups (Fig. 1A). All samples comprised predominantly a population of small EVs (30–100 nm), but also showed a small population of larger EVs (> 100 nm) probably resembling microvesicles (MVs; range > 100 up to 1000 nm, Fig. 1A). Immunoblotting results showed that oEVs were positive for known exosomal markers (CD9, ALIX, and TSG101, Fig. 1B). When small EVs were compared to large EVs (obtained from pellet after centrifugation at 12,000 xg), much stronger bands were found in small EVs samples compared to large EVs for all the markers tested (Fig. 1B). Analysis of oEVs concentration and size distribution by nanoparticle tracking analysis (NTA) revealed no significant differences in particle concentration or size distribution among experimental groups (Fig. 1C-D). However, RNA concentration was significantly higher in pregnant does compared to non-pregnant for both L and H lines (Fig. 1E) (L_P_oEV: range 69.7–296.7 ng/µl; L_NO_oEV: 0.7–10.8 ng/µl; H_P_oEV: 34.0-147.0 ng/µl; H_NO_oEV: 0.3-7.0 ng/µl). Differences were also found in RNA profiles between pregnant and non-pregnant samples (Fig. 1F) for both lines.

### RNA cargo of rabbit oEVs

A total of 4706 genes (excluding miRNAs) were identified in all experimental groups (Supplementary data S1, Table 1). Analysis of miRNAs by miRDeep2 resulted in the identification of 122 miRNAs with at least 1 count in at least 3 replicates out of four/experimental group (Supplementary data S2, Table 1). To select miRNAs for further statistical analysis, a higher threshold was used, i.e., at least 10 counts in at least 3 samples out of four/experimental group (at least in one group), resulting in 59 miRNAs (Supplementary data S2, Table 2).

**Fig. 2:**
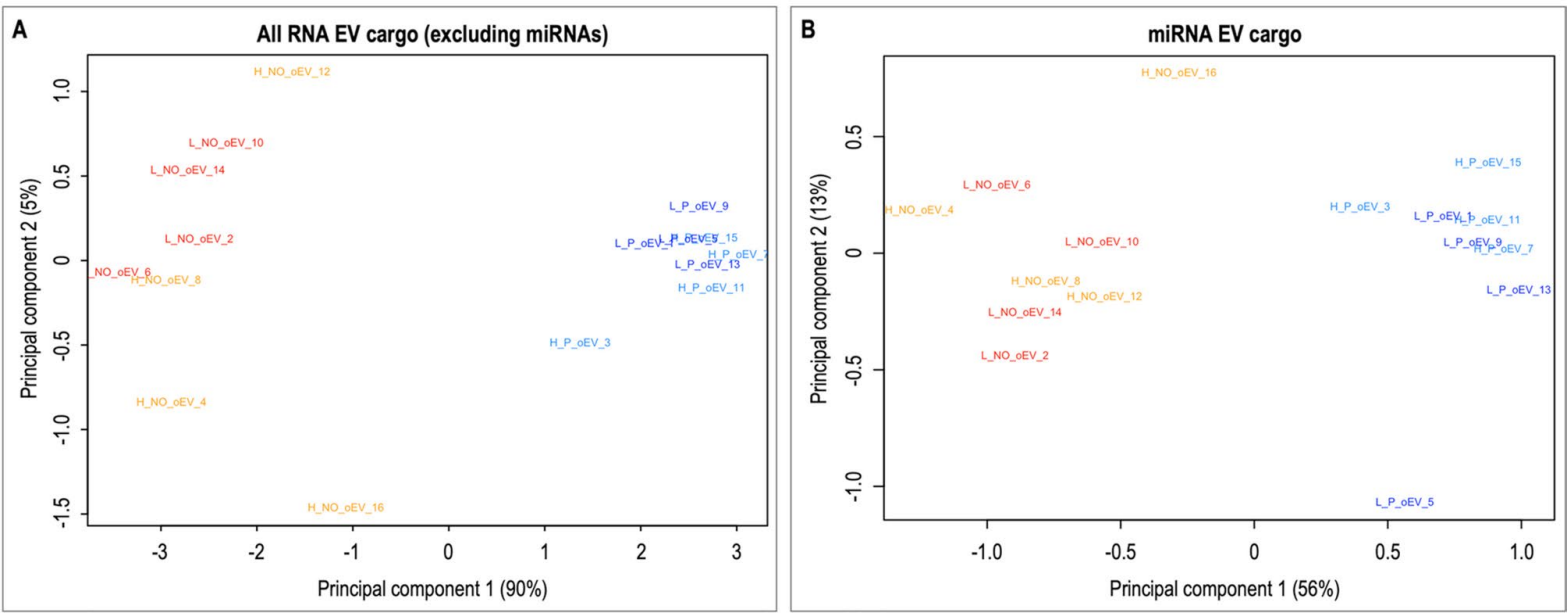
Principal Component analysis (PCA) of RNAs identified in rabbit oviductal extracellular vesicles (oEVs). (**A**) PCA of all RNA (excluding miRNAs). (**B**) PCA of microRNAs. In dark blue L_P: Line L (homogeneous litter size), pregnant samples; In light blue H_P: Line H (heterogeneous litter size), pregnant samples; In red L_NO: L line, non-pregnant samples; In light blue H_P: H line, non-pregnant samples

Principal component analysis (PCA) based on all RNAs cargo and miRNA cargo in oEVs showed a clear clustering of samples into two different groups: pregnant and non-pregnant samples for principal component 1 (Fig. 2A and B). In Fig. 2A for all RNAs, non-pregnant samples of the H group are more disperse for principal component 2 than L samples, while for pregnant does, H and L samples clustered closely together. For miRNAs, PCA (Fig. 2B) also showed a separation of samples in pregnant and non-pregnant based on principal component 1, but without differences between H and L lines for the non-pregnant groups.

### Comparison of oEVs RNA content between pregnant and non-pregnant does

Statistical analysis was performed between pregnant and non-pregnant does in separate for the total of 4706 genes and 59 miRNAs based on all annotated rabbit genes and on miRbase miRNA sequences, respectively.

#### Differential RNA cargo in oEVs between pregnant and non-pregnant does

**Fig. 3:**
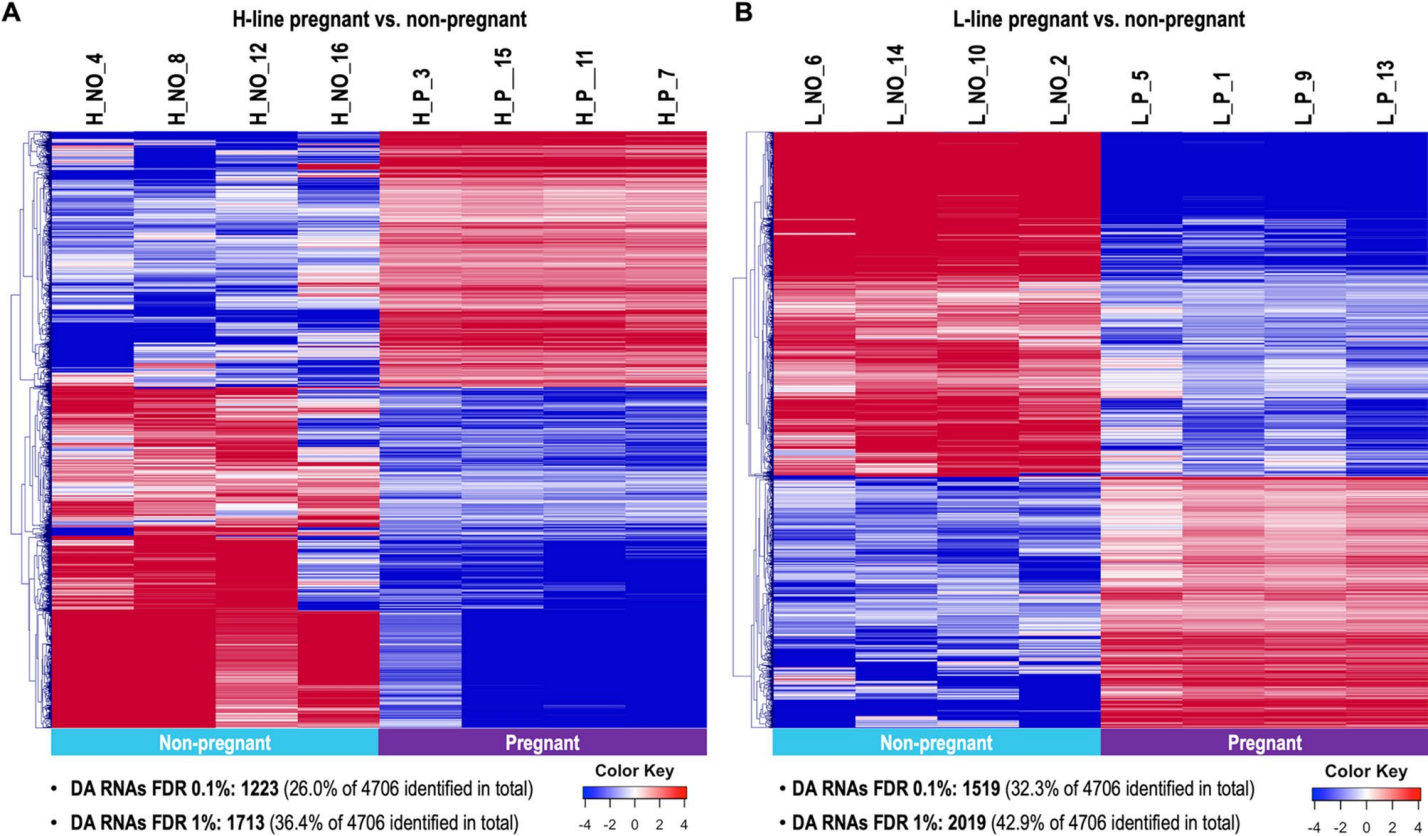
Analysis of differentially abundant RNAs in oviductal extracellular vesicles from pregnant vs. non-pregnant does. Hierarchical cluster analysis (HCL) (MeV software) was performed across samples from the H line (heterogeneous litter size) (**A**) and samples from the L line (homogeneous litter size) (**B**) for differentially abundant (DA) RNAs (FDR 0.1%). Mean-centered log2 counts per million (cpm) values were calculated (log2 of CPM of respective sample – mean of all samples)

Overall, a higher variation in RNA concentrations was observed for the H line compared to the L line for NO does (coefficient of variation 73.5% in H vs. 57.3% in L for differentially abundant (DA) RNAs at FDR 5%). Lower variability of gene expression was observed between samples of group H_P for both genes with increased expression and genes with decreased expression in pregnant does. In H line, RNAs of 1223 genes (26%) (FDR < 0.1%) were differentially abundant (DA) between pregnant and non-pregnant samples (1713 RNAs, 36.4%, for a less stringent FDR < 1%) (Supplementary data S1, Table 1). Hierarchical cluster (HCL) analysis of the 1223 DA RNAs (FDR < 0.1%) illustrates clear expression differences between pregnant and non-pregnant does in H line (Fig. 3A). Due to the high variability in the H_NO group, a self-organizing tree algorithm (SOTA, Multi Experiment Viewer software) analysis was performed resulting in 6 clusters of genes with similar expression profiles across all samples (Supplementary Fig. S1). Three of these 6 clusters (clusters 4–6) showed less variability of gene expression among samples of non-pregnant animals and were selected for further functional annotation analysis. Clusters 5 (235 genes) and 6 (290 genes) displayed genes with higher abundance in pregnant vs. non-pregnant does in H line, with strong similarities across pregnant samples and with slight variability in non-pregnant samples. Cluster 4 (414 genes) contained genes with higher abundance in non-pregnant vs. pregnant does in H line. The list of genes from each cluster can be found in Supplementary data S1, Table 2.

In L line, RNAs of 1519 genes (32.3%) were identified as DA (FDR < 0.1%) between pregnant and non-pregnant samples (2019 RNAs, 42.9%, FDR < 1%) (Supplementary data S1, Table 1). Comparison of the DA genes to the H line revealed an overlap of 978 DA genes (FDR 0.1%). Hierarchical cluster (HCL) analysis of the 1519 DA RNAs (FDR < 0.1%) showed clearly different profiles associated to pregnant and non-pregnant does in L line (Fig. 3B). Low variability of gene expression between does was observed in both P and NO groups of the L line, respectively.

#### Differential miRNA cargo in oEVs between pregnant and non-pregnant does

**Fig. 4:**
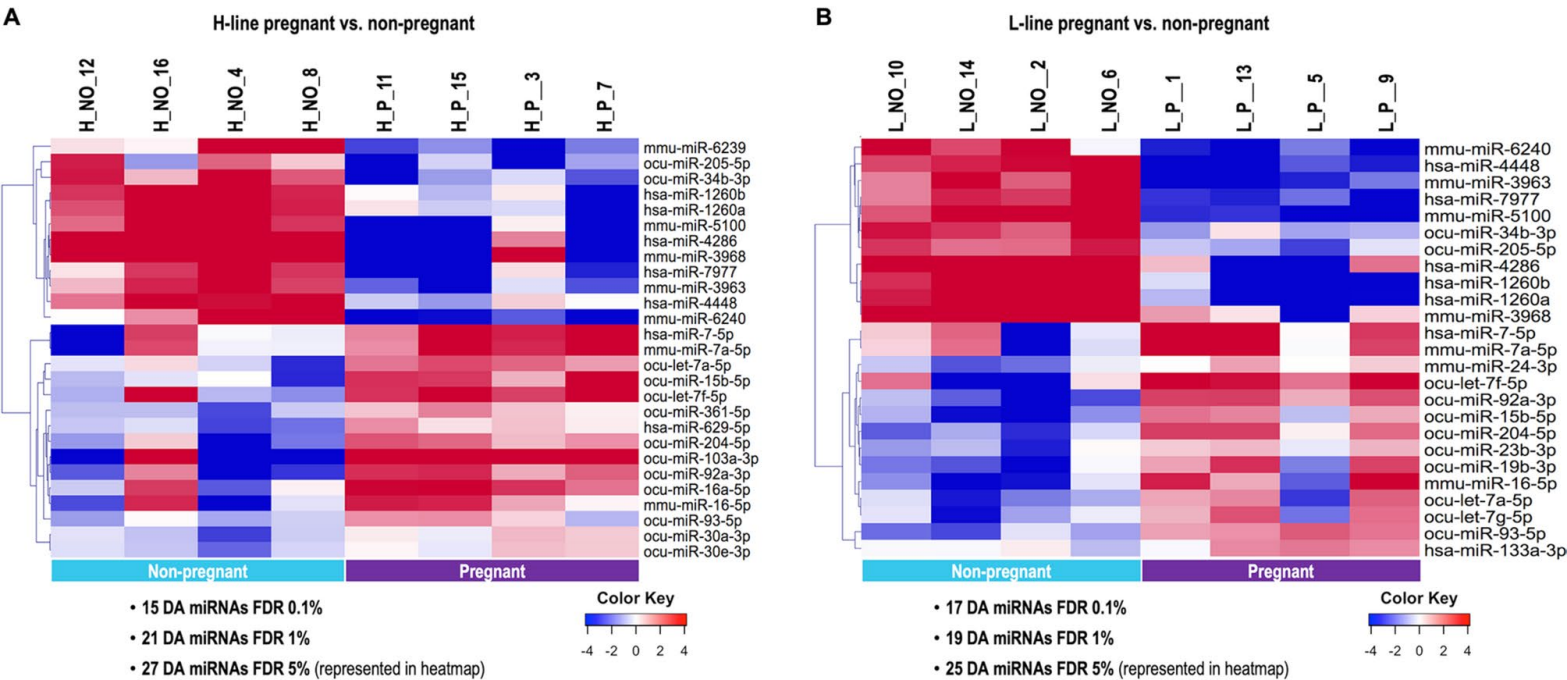
Analysis of differentially abundant microRNAs (miRNAs) in oviductal extracellular vesicles from pregnant vs. non-pregnant does. Hierarchical cluster analysis (HCL) (MeV software) was performed across samples from the H line (heterogeneous litter size) (**A**) and samples from the L line (homogeneous litter size) (**B**) for differentially abundant (DA) miRNAs (FDR 5%). Mean-centered log2 counts per million (cpm) values were calculated (log2 of CPM of respective sample – mean of all samples)

In line H, among the 59 miRNAs used for DA analysis, 27 (45.8%) (FDR < 5%) miRNAs were DA between pregnant and non-pregnant samples (21 miRNAs, FDR < 1% and 15, FDR < 0.1%) (Supplementary data S2, Table 3). HCL analysis of these 27 DA miRNAs (FDR < 5%) is shown in Fig. 4A. SOTA clustering was performed resulting in 6 clusters of miRNAs with similar expression profiles (Supplementary Figure S2 and Supplementary data S2. Table 4), Clusters 3 (9 miRNAs with higher abundance in pregnant does), 5 and 6 (each with 5 miRNAs with lower abundance in pregnant does) were selected for further bioinformatics analysis.

In line L, 25 (42.4%) (FDR < 5%) miRNAs were DA between pregnant and non-pregnant does (19 miRNAs for FDR < 1% and 17 for FDR < 0.1%) (Supplementary data S2. Table 3). HCL analysis of the 25 DA miRNAs (FDR < 5%) is shown in Fig. 4B. Comparison of the L line DA miRNAs to the H line revealed an overlap of 20 DA miRNAs (FDR 5%). Expression differences between P and NO samples were similar for the overlapping DA miRNAs. SOTA clustering resulted in 6 clusters of miRNAs with similar expression profiles (Supplementary Figure S3 and Supplementary data S2, Table 5). Clusters 3 and 5 miRNAs (4 and 2 miRNAs, respectively) showed higher abundance in P vs. NO samples, while cluster 6 (11 miRNAs) showed lower abundance in P compared to NO samples and were selected for further bioinformatics analysis.

### Comparison of oEVs RNA content between H and L lines

#### Differential RNA cargo in oEV between H and L lines

**Fig. 5:**
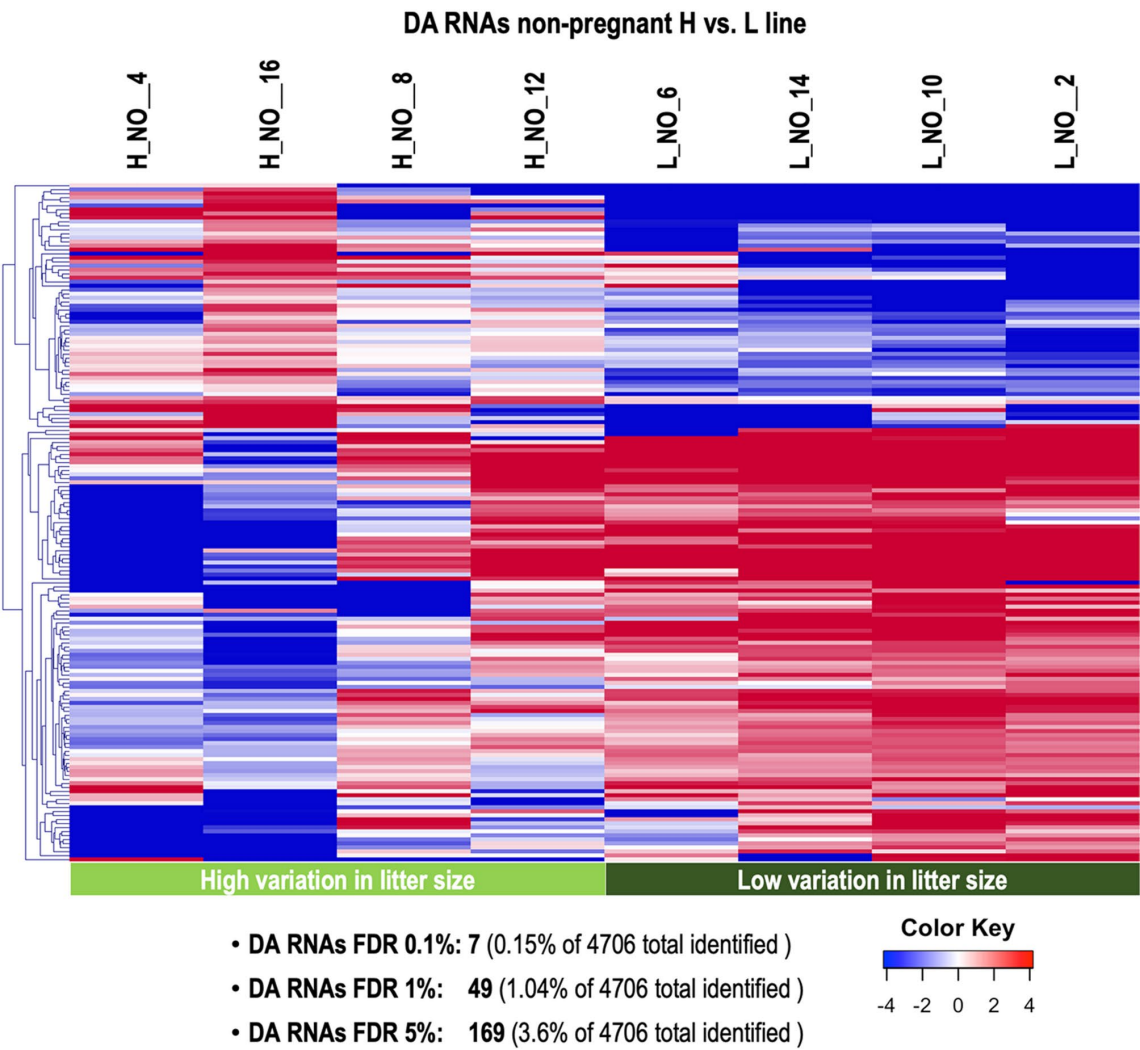
Analysis of differentially abundant RNAs in oviductal extracellular vesicles between lines in non-pregnant does. Hierarchical cluster analysis (HCL) (MeV software) was performed across samples for differentially abundant (DA) RNAs (FDR 5%). Mean-centered log2 counts per million (cpm) values were calculated (log2 of CPM of respective sample – mean of all samples)

In non-pregnant does, comparison between H and L lines identified DA RNAs of 169 genes (3.6%) (FDR 5%) (49 RNAs for FDR 1% and 7 for FDR 0.1%) (Supplementary data S1, Table 3). The comparison of these 169 DA RNAs with the DA RNAs for P vs. NO in H line and L line, respectively, revealed an overlap of 33 (H) and 116 (L). The 116 DA RNAs overlapping with the DA RNAs for P vs. NO in the L line were further checked for their log2 fold changes. All DA RNAs increased for H vs. L line in non-pregnant animals were increased for P vs. NO in the L line. Vice versa, RNAs were decreased for P vs. NO in the L line. Hierarchical cluster (HCL) analysis of the 169 DA RNAs (FDR 5%) is shown in Fig. 5. SOTA clustering analysis resulted in 6 clusters of genes with similar expression profiles (Supplementary Figure S4 and Supplementary data S1, Table 4). Three clusters with less variability of gene expression among samples were selected for further functional annotation analysis: Cluster 6 (107 genes) with higher abundance in L vs. H in non-pregnant does; Cluster 1 (39 genes), and Cluster 3 (6 genes), both with decreased abundance of RNAs in L vs. H in non-pregnant samples, which was more evident for the few genes contained in cluster 3 (Supplementary Figure S4 and Supplementary data S1, Table 4).

In pregnant does, the comparison between H and L lines at the RNA level revealed only 1 gene (*GNAO1*, G protein subunit alpha o1) (0.02%) with significant expression difference (FDR < 0.1%) (Supplementary data S1, Table 5).

#### Differential miRNA cargo in oEV between H and L lines

In non-pregnant does, no statistical differences were detected between L and H lines (Supplementary data S2, Table 6). Only miR-26a-5p had an EdgeR *P*-value of 0.018 and a Student’s t-test *P*-value of 0.017. Likewise, no statistical differences between L and H lines were obtained for pregnant does (Supplementary data S2, Table 7).

### Functional term overrepresentation analysis of DA RNAs in oEV cargo

**Fig. 6:**
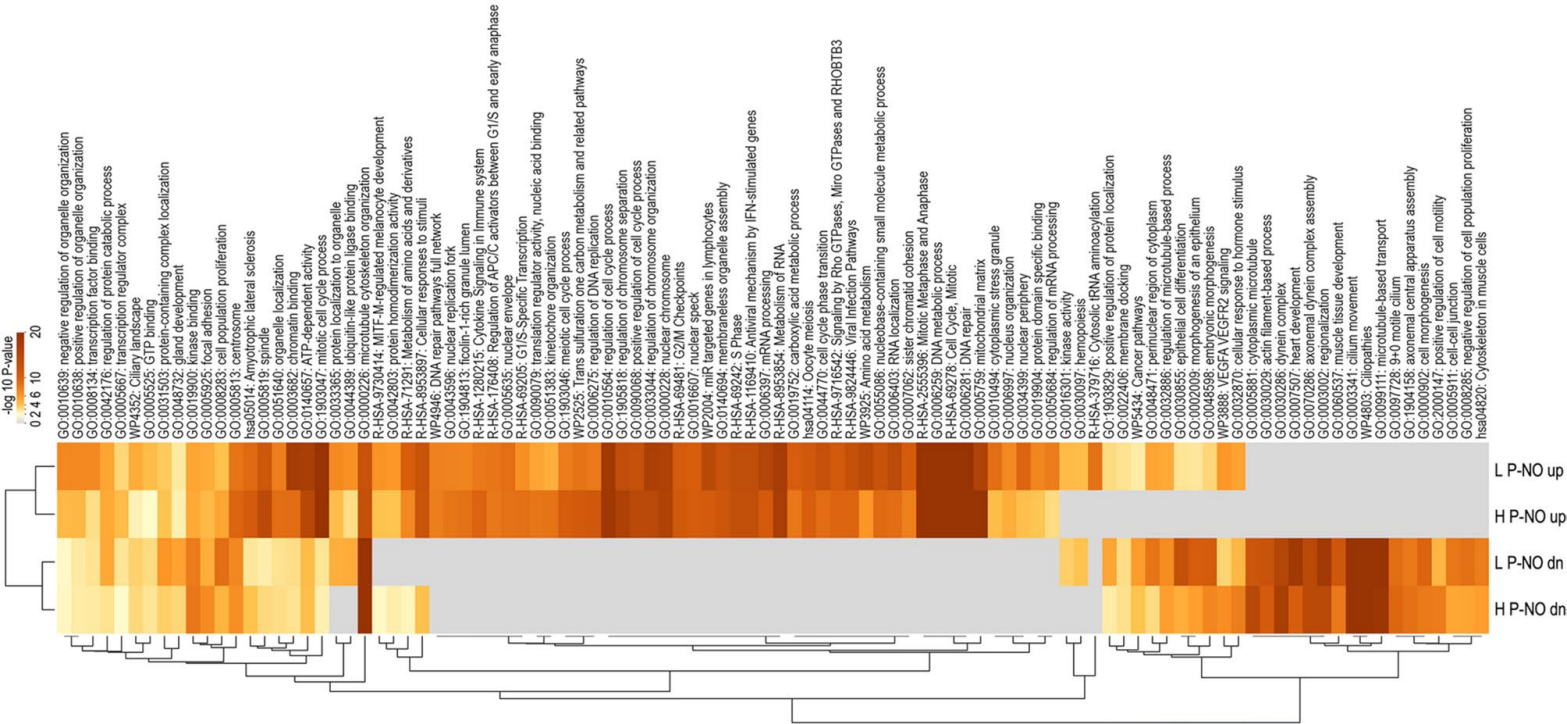
Functional term enrichment analysis for differential abundant RNAs between pregnant and non-pregnant does. Heatmap visualization of the top 100 enriched functional terms. H P-NO_up/dn: genes up/down-regulated in oEVs of pregnant vs. non-pregnant does for the H line (heterogeneous litter size); L P-NO_up/dn: genes up/down-regulated in oEVs of pregnant vs. non-pregnant does for the L line (homogeneous litter size). Heatmap of enriched terms is colored by enrichment score (–log 10 *P*-value, significant from a score of 2)

First, functional enrichment analysis was performed using Metascape to compare enrichment of functional terms and pathways associated to DA RNAs identified from the comparisons between pregnant and non-pregnant does in the H line (524 up- and 699 downregulated) and in the L line (640 up- and 879 downregulated). The top 100 overrepresented terms are illustrated in the heatmap in Fig. 6 and listed in Supplementary data S1, Table 7 together with their log 10 *P*-value for each list. Overall, similar overrepresentation of functional terms for up- and downregulated genes was observed in comparison of the two rabbit lines. The most significant functional terms for genes upregulated in oEVs of pregnant does were ‘mitotic cell cycle process’, ‘chromatin binding’, and ‘mitochondrial matrix’. In contrast, the most significant functional terms for genes downregulated in oEVs of pregnant does were ‘cilium movement’, ‘microtubule cytoskeleton organization’, and ‘epithelial cell differentiation’.

With respect to the rabbit genetic line, higher enrichment was found for functional terms such as ‘epithelial cell differentiation’, ‘chromatin binding’ (genes upregulated in P), ‘VEGFA VEGFR2 signaling’ (genes upregulated in P), and ‘Cytosolic tRNA aminoacylation’ for the L line. The terms ‘mitochondrial matrix’, ‘cilium movement’, ‘positive regulation of cell motility’, and ‘VEGFA VEGFR2 signaling’ (genes downregulated in P) were more significantly enriched for the H line.

**Fig. 7:**
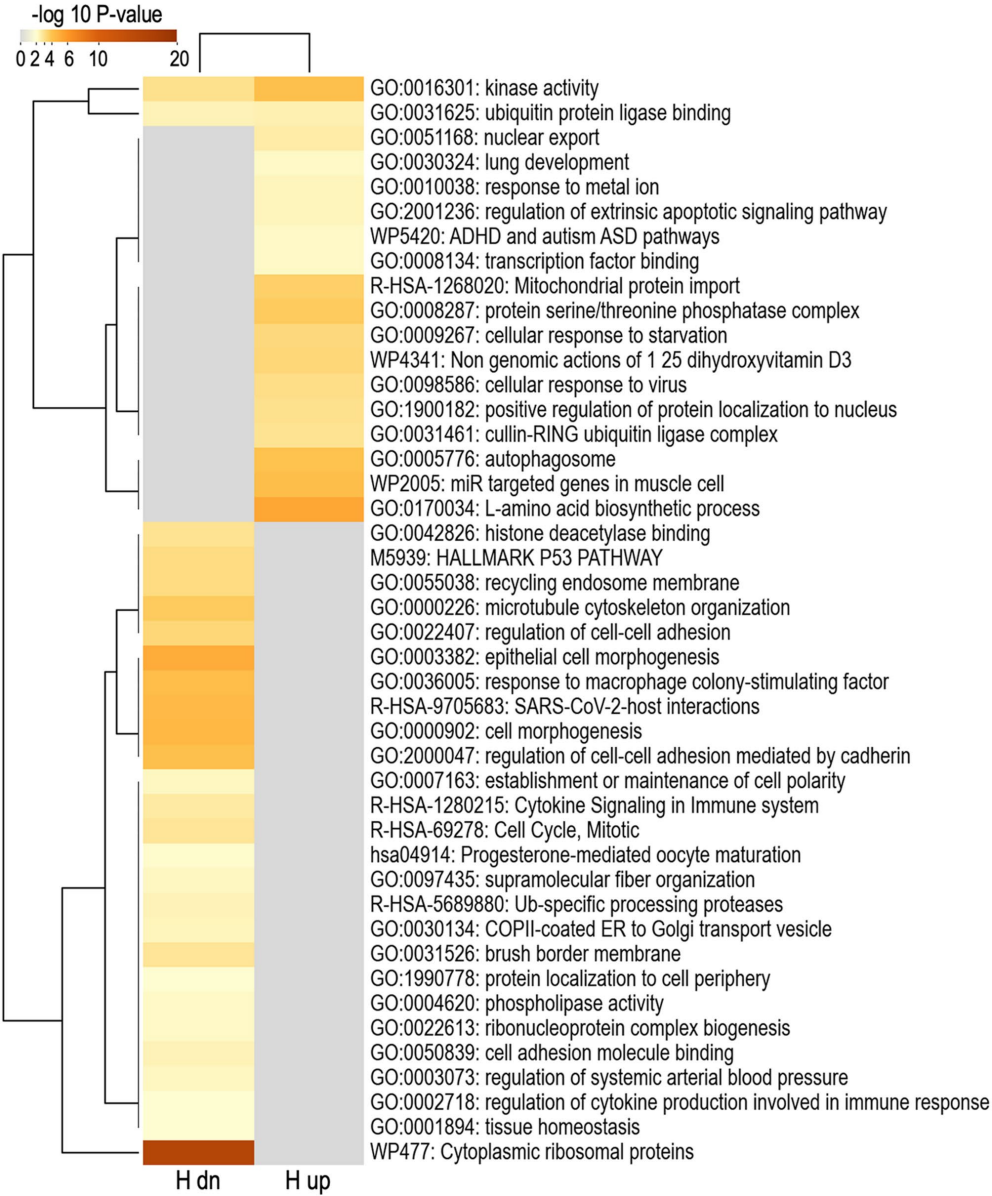
Functional term enrichment analysis for differential abundant RNAs between lines for non-pregnant does. Heatmap visualization of the top 44 enriched functional terms. H up/dn: genes up/down-regulated in oEVs of non-pregnant does for the H line (heterogeneous litter size) compared to the L line (homogeneous litter size). Heatmap of enriched terms is colored by enrichment score (–log 10 *P*-value, significant from a score of 2)

The functional term overrepresentation analysis (Metascape) for the 169 genes encoding the DA RNAs between the two rabbit lines in non-pregnant does revealed 44 significantly enriched functional terms are illustrated in the heatmap in Fig. 7 and listed in Supplementary data S1, Table 8 together with their log 10 *P*-value for each list. The most significant functional terms for RNAs with higher levels in oEVs of the H line were ‘L-amino acid biosynthetic process’, ‘kinase activity’ (also enriched for RNAs with lower levels the H line), and ‘autophagosome’. For RNAs with decreased levels in oEVs of the H line, the terms ‘cytoplasmic ribosomal proteins’, ‘epithelial cell morphogenesis’, and ‘regulation of cell-cell adhesion mediated by cadherin’ were strongly enriched.

**Fig. 8:**
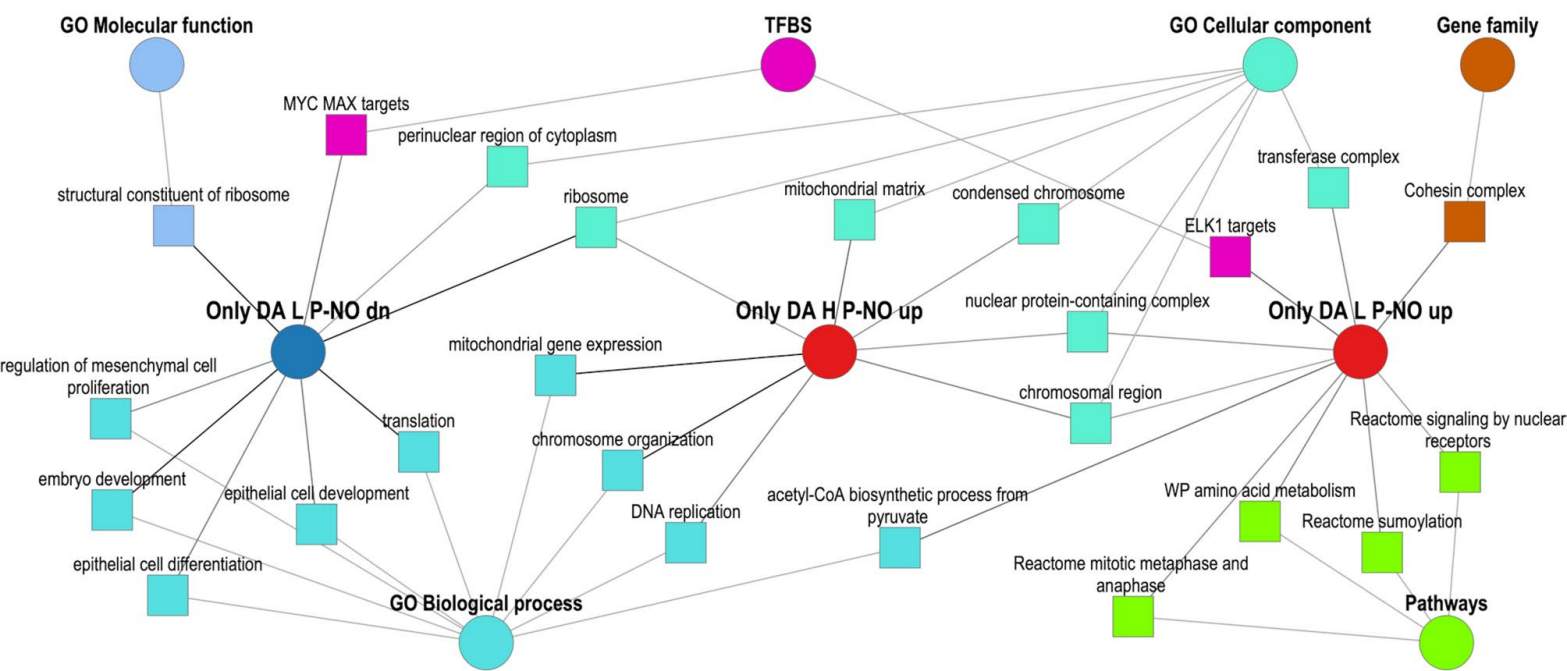
Network of overrepresented functional terms for RNAs only differentially abundant in oviductal extracellular vesicles of L line in pregnant vs. non-pregnant does. Functional annotation enrichment analysis was performed using ToppCluster. Only DA L P-NO up/dn: genes up/down-regulated in pregnant vs. non-pregnant does only in the L line (homogeneous litter size); Only DA H P-NO up: genes up-regulated in pregnant vs. non-pregnant does only in the H line (heterogeneous litter size); GO: Gene Ontology; TFBS: transcription factor binding site

To further focus on the differences between H and L lines for the RNAs significantly DA between P and NO groups, DA RNAs were compared using Venn diagrams, i.e., DA RNAs FDR 0.1% for H line with DA RNAs FDR 1% for L line and vice versa. This resulted in 144 RNAs only differential for P vs. NO in H line and 334 RNAs only differential for P vs. NO in L line (Supplementary data S1, Table 9). Results of Metascape functional term enrichment analysis for the corresponding gene lists (split into up- and downregulated genes) are shown in Fig. S5 and Supplementary data S1, Table 10). Furthermore, functional term enrichment analysis was performed with ToppCluster and a network was generated which is shown in Fig. 8. The results of the two overrepresentation analyses were similar.

For RNAs increased P vs. NO in oEVs of the H line, terms such as ‘DNA replication’, ‘mitochondrial translation elongation’, and ‘Hallmark MYC targets V1’ were specifically overrepresented. For RNAs decreased P vs. NO in oEVs of the H line, ‘cellular response to hormone stimulus’, ‘regulation of lipid metabolic process’, and ‘HIF-1 signaling pathway’ were specifically enriched. Specific functional term overrepresentation was also found for RNAs increased P vs. NO in oEVs in the L line, such as ‘acetyl-CoA biosynthetic process from pyruvate’, ‘RNA Polymerase II pre-transcription events’, and ‘regulation of peptide hormone secretion’. In addition, ‘ciliary landscape’ and ‘chromatin binding’ were enriched for RNAs increased in the L line and for RNAs decreased in the H line. Likewise, ‘Hallmark MYC targets V1’ and ‘regulation of protein modification by small protein conjugation or removal’ were enriched for RNAs increased in the H line and RNAs decreased in the L line. For RNAs specifically decreased P vs. NO in oEVs in the L line, functional terms such as ‘epithelial cell development’, ‘cellular response to cytokine stimulus’, and ‘Hallmark glycolysis’ were overrepresented. A summary of the results obtained from the different functional term overrepresentation analyses regarding the main differences between the L and H lines is shown in Table 3.

**Table 3:** Results obtained from functional term overrepresentation analyses regarding the main differences between lines

Functional term ID	Term description	H *P*-NO up	L *P*-NO up	H *P*-NO dn	L *P*-NO dn	only H *P*-NO up	only L *P*-NO up	only H *P*-NO dn	only L *P*-NO dn	NO H-L up	NO H-L dn
GO:0006086	acetyl-CoA biosynthetic process from pyruvate						-7.7*				
GO:0005776/hsa04140	autophagosome/autophagy - animal							-3.6		-4.1	
GO:0071345	cellular response to cytokine stimulus								-4.1		
GO:0032870	cellular response to hormone stimulus		-5.5	-7.8	-4.6			-5.1			
GO:0003682	chromatin binding	-9.1	-17.9	-3.0	-3.2		-5.7	-2.3			
WP4352	Ciliary landscape	-2.5	-5.1	-3.4	-3.6		-2.4	-3.2			
GO:0003341	cilium movement			-43.0	-36.1						
WP477	Cytoplasmic ribosomal proteins										-15.7
R-HSA-379,716	Cytosolic tRNA aminoacylation		-8.7				-5.4				
GO:0006260	DNA replication					-7.0					
GO:0002064/GO:0030855/GO:0003382	epithelial cell development/ differentiation/morphogenesis		-2.9	-5.0	-13.3				-8.1		-5.1
M5937	Hallmark glycolysis								-4.0		
M5926	Hallmark MYC targets V1					-4.6			-4.0		
hsa04066	HIF-1 signaling pathway							-3.0			
GO:0016301	kinase activity		-4.4		-3.7					-4.2	-3.1
GO:0170034	L-amino acid biosynthetic process									-5.3	
GO:0005759	mitochondrial matrix	-21.9	-16.3								
R-HSA-1,268,020	Mitochondrial protein import									-3.6	
R-HSA-5,389,840	Mitochondrial translation elongation					-8.3					
GO:2,000,147	positive regulation of cell motility			-8.0	-4.7						
GO:2,000,047	regulation of cell-cell adhesion mediated by cadherin										-4.2
GO:0019216	regulation of lipid metabolic process							-3.4			
GO:0090276	regulation of peptide hormone secretion						-3.3				
GO:1,903,320	regulation of protein modification by small protein conjugation or removal					-5.2			-2.0		
R-HSA-674,695	RNA Polymerase II Pre-transcription Events						-5.8				
WP3888	VEGFA VEGFR2 signaling		-6.1	-6.8	-3.5						

*Log 10 *P*-value

### Predicted target gene analysis of MiRNAs DA in oEV cargo

**Fig. 9:**
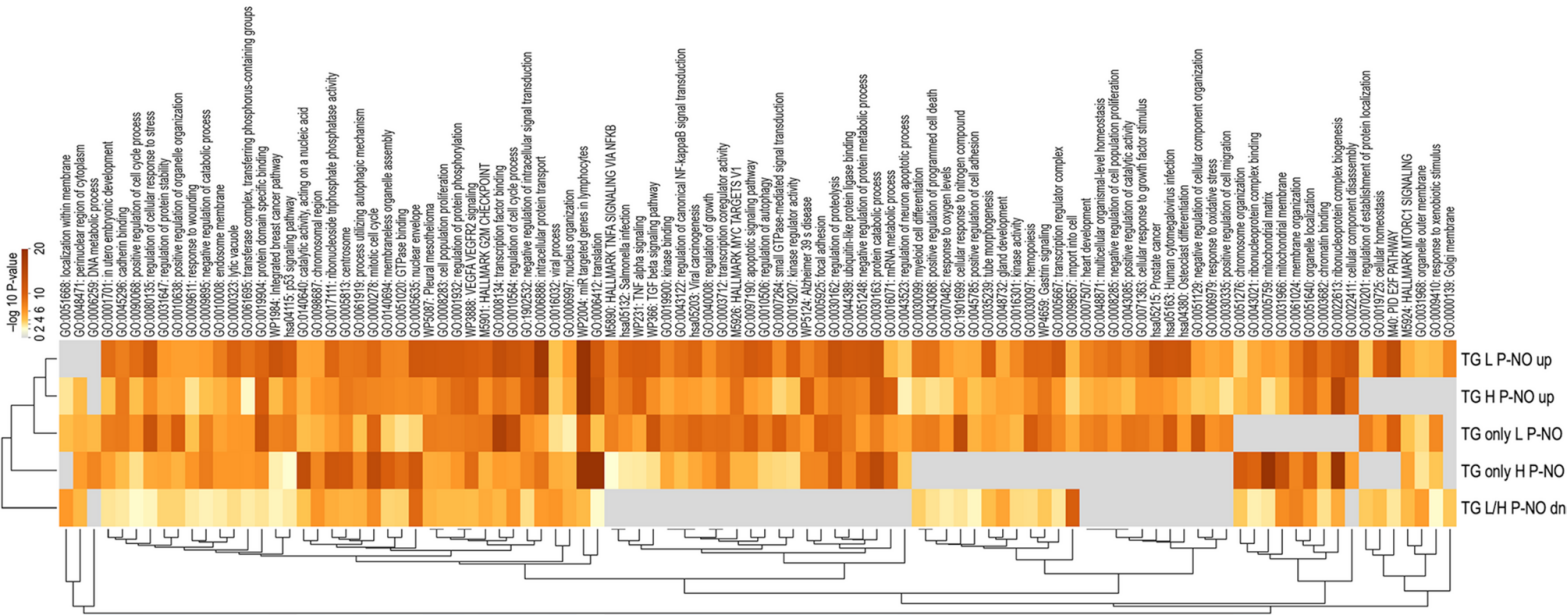
Functional term enrichment analysis for predicted target genes of differentially abundant microRNAs between pregnant and non-pregnant does. Heatmap visualization of the top 100 enriched functional terms. TG H P-NO_up/dn: target genes of miRNAs up/down-regulated in oEVs of pregnant vs. non-pregnant does for the H line (heterogeneous litter size); TG L P-NO_up/dn: target genes of miRNAs up/down-regulated in oEVs of pregnant vs. non-pregnant does for the L line (homogeneous litter size); TG only H/L P-NO: target genes of miRNAs only DA in oEVs of pregnant vs. non-pregnant does for the H or L line. Heatmap of enriched terms is colored by enrichment score (–log 10 *P*-value, significant from a score of 2)

Predicted target genes (TGs) analysis was performed for the miRNAs increased or decreased between P and NO in oEVs of both rabbit lines. Furthermore, TGs analysis was also performed for the miRNAs only DA between P and NO in the H line or the L line (Supplementary data S3, Table 1). The lists of predicted TGs together with the corresponding miRNAs and statistical values are shown in Supplementary data S3, Tables 2, 3, 4, 5 and 6. These 5 lists (downregulated miRNAs in oEVs of L and H line were the same) of predicted TGs were used for a comparative functional term enrichment analysis with Metascape. Figure 9 shows a heatmap of the top 100 overrepresented functional terms for these 5 lists of miRNA TGs (top 100 terms together with their log 10 *P*-value for each list are shown in Supplementary data S3, Table 7). A summary of the results obtained from the miRNA target gene analysis and functional term overrepresentation analysis of target genes regarding the main differences between the L and H lines is shown in Table 4.

**Table 4:** Summary of the results obtained from functional term overrepresentation analysis of MiRNA target genes

Term ID	Term description	L *P*-NO up	H *P*-NO up	L/H *P*-NO dn	only L *P*-No	only H *P*-No
M40	PID E2F pathway (E2F transcription factor network)	-14.1*		-2.9	-10.1	
GO:0008134	transcription factor binding	-13.5	-8.4	-4.6	-16.7	-5.8
GO:0035239	tube morphogenesis	-9.9	-5.0	-4.3	-5.6	
GO:0071363	cellular response to growth factor stimulus	-8.4	-6.4		-8.3	
WP366	TGF beta signaling pathway	-10.1	-6.7		-12.0	-3.1
WP4659	gastrin signaling	-9.4	-8.3	-3.9	-8.8	
M5924	Hallmark MTORC1 signaling	-5.0		-4.5	-4.0	-5.4
hsa04115	p53 signaling pathway	-10.5	-6.8	-2.8	-8.0	-2.1
GO:0097190	apoptotic signaling pathway	-11.0	-5.8		-9.9	-4.8
GO:0007264	small GTPase-mediated signal transduction	-7.0	-3.4		-10.1	-3.4
GO:0019207	kinase regulator activity	-9.9	-3.9		-7.7	-3.2
WP2004	miR targeted genes in lymphocytes	-29.3	-23.5	-4.4	-10.6	-26.9
GO:0080135	regulation of cellular response to stress	-12.2	-6.8	-2.4	-12.5	-5.3
GO:0006979	response to oxidative stress	-5.2	-4.0		-6.4	
GO:0051276	chromosome organization	-3.4	-4.3	-3.8		-10.5
GO:0010564	regulation of cell cycle process	-12.2	-7.4	-6.4	-13.7	-5.7
GO:0019725	cellular homeostasis	-9.7		-4.2	-5.9	
GO:0005635	nuclear envelope	-11.5	-7.0	-9.4	-3.4	-11.5
GO:0000139	Golgi membrane	-7.1		-3.9		
GO:0061024	membrane organization	-8.1	-7.1	-7.5		-7.4
GO:0070201	regulation of establishment of protein localization	-3.7		-3.8	-7.9	
GO:0030335	positive regulation of cell migration	-5.7	-2.9		-7.3	
GO:0005759	mitochondrial matrix	-5.7	-3.0	-2.9		-21.1
GO:0031966	mitochondrial membrane	-5.3	-4.0	-6.8		-13.2
GO:0043021	ribonucleoprotein complex binding	-5.1	-5.1	-2.5		-9.6
GO:0006412	translation	-11.4	-12.5	-2.1	-4.1	-29.4

L P-NO up: target genes of miRNAs upregulated P vs. NO in the L line; only L P-No: target genes of miRNAs only DA P vs. NO in the L line; *Log 10 *P*-value

## Discussion

This study provides so far with the first molecular signature of rabbit oEVs at RNA level, with a clear differential RNA cargo in oEVs from pregnant vs. non- pregnant does and more interestingly, between two rabbit lines selected for high and low (H and L) litter size variability. The results point at the oEVs as key contributors in the embryo-maternal crosstalk and supporting embryo development in rabbits as in other species [22, 58]. Moreover, the results suggest that the oEVs’ molecular content in the L line might contribute to a more supportive oviductal milieu for embryo development compared to the H line, and thus, providing a higher number of embryos and pregnancy rates.

The does used in this study belong to a breeding program, in which two rabbit lines were divergently selected for litter size variability over 12 generations and therefore representing a unique material for investigation [5]. As a result of this successful selection progress [5], the litter size variability was lower and average litter size higher in the L line in contrast to the H line. The litter size variability in the females chosen for this experiment was 3.45 kits² for the L line and 6.13 kits² for the H line. These values are representative of the 12th generation to which they belong, where the overall variability was 2.27 kits² for the L line and 3.84 kits² for the H line.

In previous studies, we reported that the higher litter size of the L line compared to the H line was not due to differences in ovulation rate, as both lines have similar ovulation rates. However, the L line shows a higher number of normal embryos and better embryonic development at 72 h post-coitum than the H line. These differences are maintained until 12 days of gestation and parity [8, 9]. Another parameter of interest is the difference between the lines based on pregnancy status, as it has been confirmed that the L line exhibits a lower fertility percentage. Fertility percentage is defined as the proportion of females culled due to infertility after either four consecutive non-fertile matings or seven consecutive male rejections [6]. Thus, differences in the litter size and fertility might be due to differences in the oviductal milieu and the results of the present study show that at least in part this could be due to differences in oEV secretions and the molecular cargo between the two lines.

The characterization of oEVs showed a population of EVs with size rage similar to other species [59–62]. The oEVs concentration and size distribution did not vary between reproductive stages (pregnant vs. non-pregnant). However, pregnant does presented a much higher RNA concentration in oEVs and also different RNA profiles compared with non-pregnant does, regardless the line. Similar results have been obtained in the bovine oviductal fluid, with no differences in EVs size across different stages of the reproductive cycle [30], but with marked differences in the RNA and protein cargo due to a strong hormonal regulation [63]. Also, in equine uterine EVs, a fine-tuned regulation of the RNAs and proteins was reported by the day of pregnancy, the estrous cycle, and even the size of the embryo [47].

The high numbers of DA genes identified between P vs. NO does in the H and the L line demonstrated the marked hormonal modulation of pregnancy and the presence of embryos on oEVs protein-coding RNA cargo in does. Moreover, the DA miRNAs identified between P vs. NO does in H and L line showed that the small RNA cargo of rabbit oEVs was also regulated in a different manner in P vs. No but also between H and L lines. Our results are in line with previous studies showing that the oEVs´ molecular cargo is very dynamic and their protein-coding RNA, small non coding RNA, protein, and metabolite components change during the reproductive cycle and pregnancy to support the different events taken place in the oviduct (from gamete transport, fertilization to early stages of embryo development) [30, 64].

Regarding the differences in protein-coding RNAs cargo, one striking finding was that despite similar differences between P and NO does in the two lines, the group H NO showed a high variability between the biological replicates whereas variability within H_P and the P and NO groups of the L line was low. Interestingly, the direct comparison of the lines only showed significant differences for the NO samples, but not for pregnant does. This suggests that the high variation in litter size might be mainly related to the differentiation status of the oviduct epithelium in the NO does, and once the doe is pregnant, the changes in the oviduct might be more similar between lines than in non-pregnant does. These differences in NO does from the lines could provide insights into the observed variation in fertility between the lines. The results from the functional term enrichment analysis of the genes only DE in P vs. NO in does from in L line or only in H line also pointed at a disturbance in cellular homeostasis. While in the H line the enriched GO terms were more related to cell functionality (organelles: mitochondria, ribosomes, metabolism of amino acids, DNA replication), for the L line, functional categories related to epithelial cell development (down-regulated in P), chromatin binding, and ciliary landscape were more enriched. The overrepresented functional terms obtained from the direct comparison of the lines (NO does) also supported an involvement of mitochondria, autophagy, and epithelium development.

Among the DA mRNAs between P vs. NO in both lines we would like to highlight the high concentration of oviductal glycoprotein 1 (*OVGP1*) mRNA in oEVs of NO does. The *OVGP1* mRNA is encoding one of the most abundant proteins in the oviduct and is strongly modulated during the oestrus cycle with highest concentration in estrus [65]. The OVGP1 protein can bind to sperm and oocytes and exert positive effects on both sperm and oocytes, including sperm capacitation, sperm motility and viability, sperm–egg binding, penetration rate and fertilization rate, decrease in polyspermy, embryo quality, and early embryo development [66]. In the rabbit, the analysis of polymorphisms in the *OVGP1* gene revealed an association with litter size [67]. High abundance of *OVGP1* transcript was also found in EVs from the bovine and porcine oviduct [25, 26, 30]. In the pig, Alcantara-Neto et al. [25] showed that oEVs carry OVGP1 protein, interact with both the cumulus–oocyte complex and spermatozoa delivering OVGP1 into the ooplasm and increasing sperm survival. Despite a similar downregulation in P does (due to the drop of estradiol after ovulation and rise of progesterone) with a log 2 fold-change of 6 and 7 in both lines, the variability of the expression levels in H NO does (approx. 16-fold lower expression in 2 out of 4 does) appeared to be higher, which needs to be validated in a higher number of biological replicates. Another interesting mRNA was *MYH9*, encoding the OVGP1 protein binding partner in gametes [68], which was also detected in bovine and porcine oEV samples [9, 27, 30]. An increased variation of the expression levels in H NO does was found similar to *OVGP1*.

Focusing on mRNAs DA between L and H line in NO does, a number of interesting genes associated to oviduct epithelium and embryo development was found as DA between lines in NO does, such as *FZD3*, *ASF1A*, *IHH*, *SLC34A2*, *BCOR*; *TIE1*, *ST14*, and *MMP15*. The most striking difference between H and L line was found for frizzled class receptor 3 (*FZD3*) mRNA with a 377-fold up-regulation in oEVs of P does in the L line, but no difference for the H line. In the H line, *FZD3* was already at the same high expression level in oEVs of NO H line does as in P does of both lines. Correspondingly, two other members of the WNT/β-catenin (WNT/CTNNB1) signaling pathway, Wnt family member 7 A (*WNT7A*) and smoothened, frizzled class receptor (*SMO*) were only downregulated in oEVs of P does of the L line and had already decreased levels in oEVs of NO does in the H line. This pathway was found altered in the oviduct in mice, strongly regulated by estrogen, and related to embryo transport [69]. Furthermore, a role of WNT signaling in oviduct epithelium development is known from domestic animal oviductal organoid models [70]. Another gene with an expression pattern very similar to *FZD3* was anti-silencing function 1 A histone chaperone (*ASF1A*), which has been described as involved in cellular reprogramming into pluripotent cells [71] and as important for pre-implantation embryonic development in mice [72].

Regarding Indian hedgehog (IHH), which plays an active role in progesterone hedgehog signaling pathway, it has been shown to promote the peri-implantation development of embryos in mice as a major mediator of progesterone signaling [73, 74] and its ablation in the murine uterus led to infertility in female mice [75]. However, in the rabbit oviduct *IHH* was down-regulated in P does but only in the L line (already low levels in oEVs of NO does in the H line). Solute carrier family 34 member 2 (*SLC34A2*), which also showed highest expression levels in oEVs of NO does in the L line and decreased levels in the L line, has been shown to be essential for early embryonic development and involved in embryonic genome activation (EGA) [76, 77]. Moreover, dynamic changes in gene expression of *IHH*, *BCOR* (BCL6 corepressor), *ST14* (ST14 transmembrane serine protease matriptase), and *MMP15* (matrix metallopeptidase 15) were reported in in uterine luminal epithelial cells during the peri-implantation period in the mouse [78]. Overall, the functional term overrepresentation analysis of the differentially expressed transcripts in oEVs between L and H line indicate a disturbed oviductal milieu in the H line related to inadequate differentiation status of the oviduct epithelium interfering with support of gamete maturation and early embryo development. These disturbances might be at least in part the reason for the higher variation in litter sizes in the H line.

Regarding the oEVs’ miRNA cargo, many of the miRNA identified as DA between P and NO does have been reported to play a role in the regulation of early embryo development [79]. Many of the miRNAs DA between P and NO were common to both lines, but a number of miRNAs were found as DA only in the H or the L line. These miRNAs could be related to the differences in embryo development support between rabbit lines. The main differences in the predicted target genes of miRNAs specifically DA for the two lines were also related to cellular homeostasis, response to stress, growth factor signalling, and apoptotic processes. Among the miRNAs DA between P and NO in both lines, different members of let-7 family (let-7a-5p, let-7f-5p, let-7 g-5p) were found, which have a conserved role in cell fate determination in the early embryo [80]. Additionally, miR-34b-3p, related to prostaglandin secretion and response to prostaglandin was identified in rabbit oEVs as downregulated in P vs. NO in both lines. Similarly, miR-34b-5p was identified in equine uterine EVs as downregulated in pregnant vs. control mares on day 13, which could result in the upregulation of genes related to embryo development. Furthermore, deficiency of miRNA clusters miR-34b/c in the murine oviduct leads to lack of cilia, resulting in failure of oocyte pick-up by the infundibulum and reduced efficiency of sperm migration and transport of embryos to the uterus [81]. MicroRNA miR-34b-3p has also been reported as upregulated in bovine oEVs collected a few days after ovulation compared to the rest of the days of the cycle [82]. Another microRNA with reduced levels in oEVs of P does in both lines was miR-205-5p, has been described as down-regulated in oviduct epithelial cells of cows pregnant 5 days after induction of ovulation [83] and as important for oocyte-to-embryo transition in the pig [84]. Injection of an ssc-miR-205 inhibitor into porcine oocytes reduced their ability to support development to the blastocyst stage by 50% compared to water-injected controls [84]. Thus, it has been suggested that ssc-miR-205 could play a role in shaping the zygotic expression or contributing to maternal mRNA degradation.

Among the miRNAs only identified as DA in P vs. NO in the L line, we would like to highlight miR-23b-3p, which has been found with increased expression in fertile human endometrium during the time of receptivity [85]. MicroRNAs miR-23b-3p and miR-24-3p, both up-regulated in oEVs of P does in the L line, have been found as increased in bovine uterine EVs, suggesting a role in regulation of embryo lipid metabolism and implantation [86]. Among the miRNAs only DA between P and NO does of the H line, miR-16-5p expression in follicular fluid has been associated with embryo quality [87]. Furthermore, high expression of miR-103a-3p in follicular fluid, which was 39-fold increased in P vs. No does in the H line, resulted in a poor quality of human embryo on days 3 and 5 during IVF treatment [88]. In support of the results from the expression of protein-coding RNAs, the differences between lines in oEVs’ miRNAs suggests a role of dysregulated oEVs’ miRNAs in differences in litter size between the rabbit lines.

Altogether, the RNA cargos of oEVs associated to embryo development, homeostasis, hormone response and cilium movement in L line, with the fact that higher pregnancy rates and more homogeneous litter size were obtained in L line, support the hypothesis that oEVs from L line might provide with a more optimal oviductal milieu facilitating embryo development. Differences in embryo development begin subtly at 48 hpc when embryos are in the ampulla and become entrenched at 72 hpc when embryos are in utero. These results are in line with previous studies showing that the use of oEVs as supplements in IVP systems improved embryo development (in cow [29, 89]); in pig [27], ; in human [90]). In pigs, oEV supplementation during only the first two days of in vitro culture showed beneficial effects on embryo development with increased cleavage and blastocyst rates compared to control group [28]. In bovine, the use of oEVs during the complete in vitro culture (7 days) enhanced blastocyst yield, quality, and embryo survival over times [27]. Lopera-Vasquez et al. [91] also observed an improvement of embryo cryosurvival when oEVs were used during complete in vitro culture in bovine, although they did not find any increase in blastocyst yield. This together with the results of our study suggest that the use of oEVs as supplements in ARTs could also be extrapolated to applications in rabbits. Furthermore, our data brings additional value to the current literature, showing that the effect of oEVs is dependent on the animal their genetic background, at least in rabbits.

## Conclusion

The study revealed three main findings: (1) Divergent selection for litter size variability affects the RNA cargo in oEVs, particularly protein-coding RNAs and miRNAs, favouring both embryonic development and survival in the line selected for low variation in litter size over the line selected for high variation. (2) In each line, the studied cargo changes between cyclic and pregnant does, suggesting a fine-tuned supporting role of oEVs in the physiological processes occurring within the rabbit oviduct. (3) The direct comparison of lines only provided differences in the non-pregnant does, indicating that once the doe is pregnant the oviductal gene expression might be more similar between lines than in non-pregnant does and providing novel insights to understand the fertility variations between the lines. Altogether, these findings show for the first time that the molecular cargo of oEVs in rabbits is remarkable dynamic.

## Supplementary Information

Below is the link to the electronic supplementary material.

Supplementary Material 1

Supplementary Material 2

Supplementary Material 3

Supplementary Material 4

Supplementary Material 5

Supplementary Material 6

Supplementary Material 7

Supplementary Material 8

## Data Availability

RNA-Seq data have been deposited at NCBI’s Sequence Read Archive (SRA), BioProject accession number PRJNA1209703 (http://www.ncbi.nlm.nih.gov/bioproject/1209703). Further data supporting the findings of this study are available from the corresponding author upon reasonable request.

## Abbreviations

DA: Differentially abundant
DEGs: Differentially expressed genes
EGA: Embryonic genome activation
EVs: Extracellular vesicles
FDR: False discovery rate SRA: sequence read archive
FGCZ: Functional Genomics Center Zurich
HCL: Hierarchical cluster
hpc: Hours post-coitum
IHH: Indian hedgehog
IVP: In vitro embryo production
MVs: Microvesicles
NTA: Nanoparticle tracking analysis
OVGP: Concentration of oviductal glycoprotein
PCA: Principal component analysis
SDS-PAGE: Sulphate-Polyacrylamide gel electrophoresis
TEM: Transmission electron microscopy
TGs: Predicted target genes
UC: Ultracentrifugation
