# Supplementary material for: Divergent selection for litter size variability affects RNA cargo in oviductal extracellular vesicles related to embryonic development and survival
Source: Biol Res. 2025 Sep 26;58:63. doi: 10.1186/s40659-025-00642-1 (PMC12465832; doi:10.1186/s40659-025-00642-1)
Supplement: Supplementary file 4 — Supplementary Material 4 [file 40659_2025_642_MOESM4_ESM.docx]

**
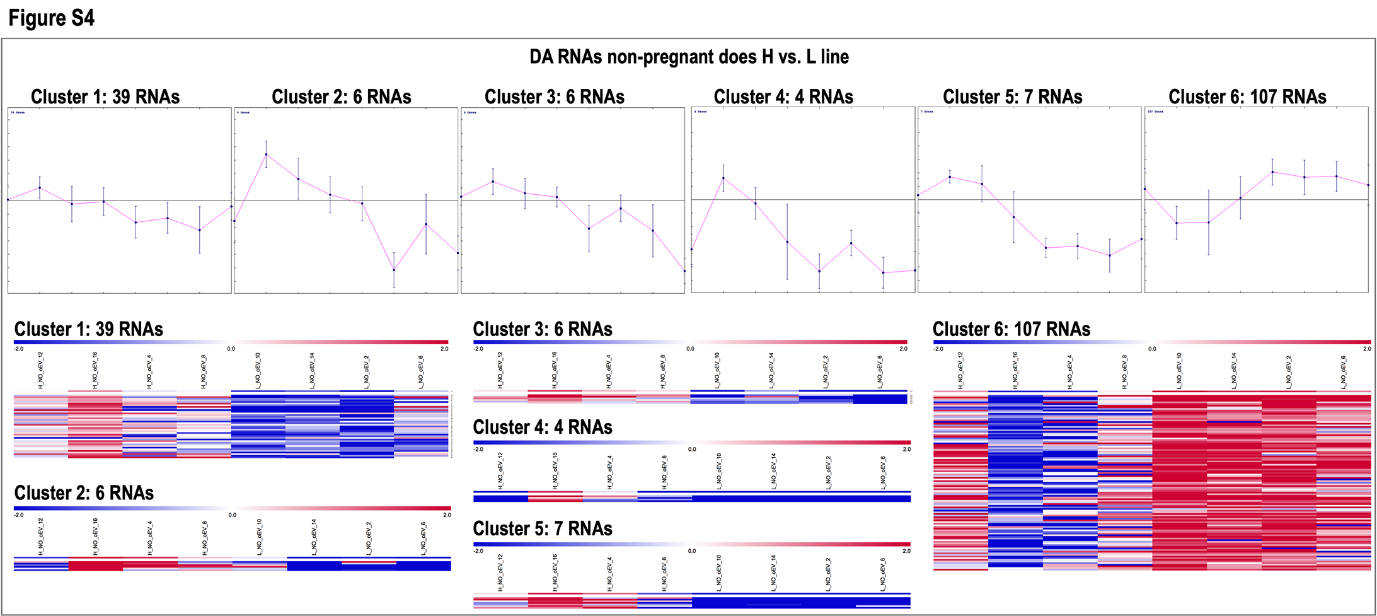
**

**Figure S4.** Self-organizing tree algorithm (SOTA, Multi Experiment Viewer software) analysis used to identify similar expression profiles across experimental groups in differential abundant (DA) RNAs derived from the H line (heterogeneous litter size) vs. the L line (homogeneous litter size) in non-pregnant does.
