# Supplementary material for: Divergent selection for litter size variability affects RNA cargo in oviductal extracellular vesicles related to embryonic development and survival
Source: Biol Res. 2025 Sep 26;58:63. doi: 10.1186/s40659-025-00642-1 (PMC12465832; doi:10.1186/s40659-025-00642-1)
Supplement: Supplementary file 5 — Supplementary Material 5 [file 40659_2025_642_MOESM5_ESM.docx]

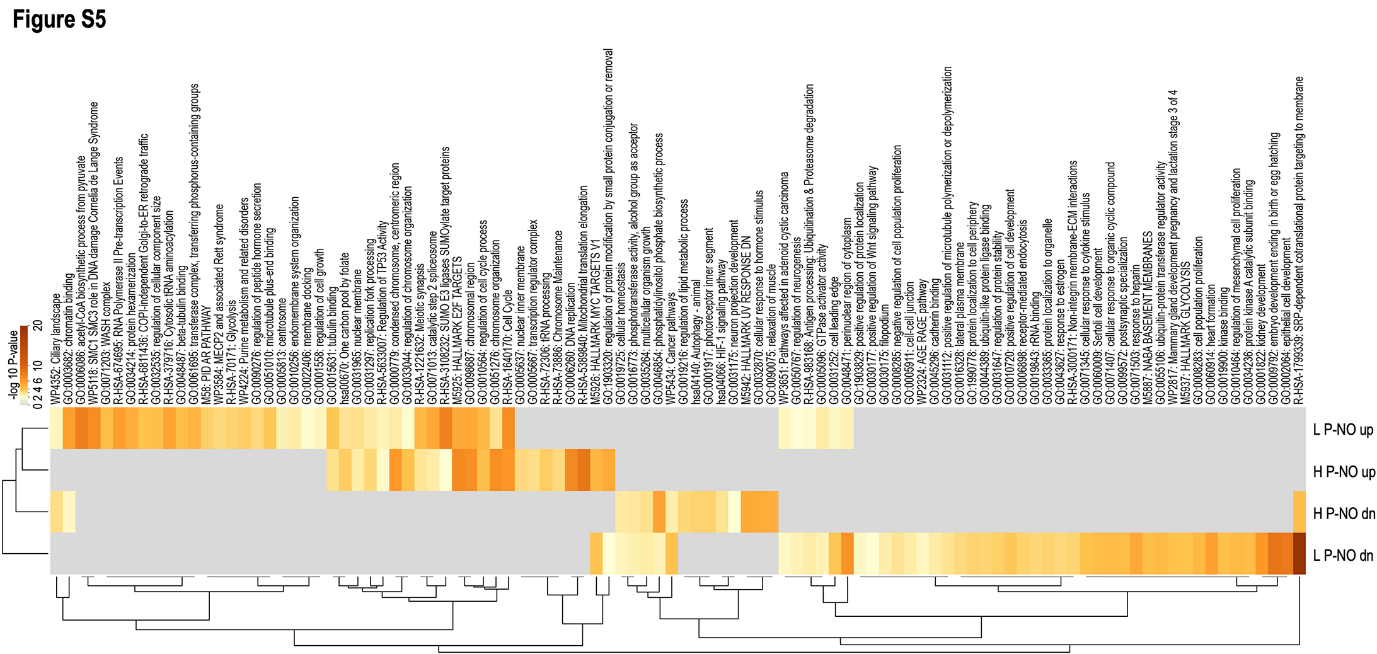


**Figure S5.** Top 100 overrepresented functional terms of the comparative Metascape analysis for RNAs only differentially abundant in oviductal extracellular vesicles (oEVs) of one of the two rabbit lines in pregnant vs. non-pregnant does.
